# Impact of patent ductus arteriosus on non-invasive assessments of lung fluids in very preterm infants during the transitional period

**DOI:** 10.1007/s00431-023-05106-w

**Published:** 2023-07-17

**Authors:** Silvia Martini, Italo Francesco Gatelli, Ottavio Vitelli, Francesca Vitali, Francesca De Rienzo, Roberta Parladori, Luigi Corvaglia, Stefano Martinelli

**Affiliations:** 1 Neonatal Intensive Care Unit, IRCCS AOU S. Orsola, Bologna, Italy; 2 Department of Medical and Surgical Sciences, University of Bologna, Bologna, Italy; 3 Division of Neonatology and Neonatal Intensive Care Unit, ASST Grande Ospedale Metropolitano Niguarda, Milan, Italy

**Keywords:** Preterm infants, Patent ductus arteriosus, Lung fluids, Lung ultrasound score, Transthoracic electrical bioimpedance, Electrical cardiometry

## Abstract

This prospective observational study aimed to evaluate whether lung fluids, assessed by lung ultrasonography and transthoracic electrical bioimpedance (TEB), may be influenced by the presence of a haemodynamically significant patent ductus arteriosus (hsPDA) in very preterm infants during the transitional period. Infants < 32 weeks of gestational age (GA) admitted to the neonatal intensive care units of IRCCS AOU Bologna and Niguarda Metropolitan Hospital of Milan (Italy) underwent a daily assessment of a lung ultrasound score (LUS) and of a TEB-derived index of thoracic fluid contents (TFC) during the first 72 h after birth. Echocardiographic scans were simultaneously performed to evaluate the concomitant ductal status (hsPDA vs. restrictive or closed duct). The correlation between LUS, TFC, and the ductal status was tested using generalized estimating equations. Forty-six infants (median GA: 29 [interquartile range, IQR: 27–31] weeks; median birth weight: 1099 [IQR: 880–1406] g) were included. At each daily evaluation, the presence of a hsPDA was associated with significantly higher LUS and TFC compared with a restrictive or closed ductus (*p* < 0.01 for all comparisons). These results were confirmed significant even after adjustment for GA and for the ongoing modality of respiratory support.

* Conclusion*: Even during the first 72 h of life, the presence of a hsPDA determines a significant increase in pulmonary fluids which can be non-invasively detected and monitored over time using lung ultrasonography and TEB.

**Table:** 

**What is Known:** • *Lung ultrasonography provides a non-invasive assessment of lung fluids and is widely used in neonatal settings.* • *In preterm infants, the persistence of a haemodynamically significant patent ductus arteriosus (hsPDA) over the first weeks can negatively affect pulmonary outcomes.*	
**What is New:** • *The presence of aan hsPDA is associated with increased lung fluids since early postnatal phases.* • *Lung ultrasonography and transthoracic electrical bioimpedance can effectively monitor lung fluid clearance in preterm infants with a hsPDA during the transitional period, with potential clinical implications.*

**What is Known:**

• *Lung ultrasonography provides a non-invasive assessment of lung fluids and is widely used in neonatal settings.*

• *In preterm infants, the persistence of a haemodynamically significant patent ductus arteriosus (hsPDA) over the first weeks can negatively affect pulmonary outcomes.*

**What is New:**

• *The presence of aan hsPDA is associated with increased lung fluids since early postnatal phases.*

• *Lung ultrasonography and transthoracic electrical bioimpedance can effectively monitor lung fluid clearance in preterm infants with a hsPDA during the transitional period, with potential clinical implications.*

## Introduction

The reabsorption of lung fluids is crucial for the respiratory adaptation to extrauterine life. Together with the primary surfactant deficit, fluid retention in the alveoli ensuing from the immaturity of the related clearance systems is a major contributor to the development of respiratory distress syndrome (RDS) in preterm neonates.

The presence of a haemodynamically significant patent ductus arteriosus (hsPDA) with an excess left-to-right transductal shunting is frequent among very preterm infants during early postnatal phases. Consistently with the resulting pulmonary overflow, which contributes to the accumulation of alveolar fluids, hsPDA has been associated with a fourfold increase of RDS risk [1].

Monitoring lung fluid clearance in preterm infants with RDS and hsPDA may provide potentially useful information for their therapeutic management. Lung ultrasonography is widely used for non-invasive assessment of lung fluids in preterm neonates [2]. Recently, transthoracic electrical bioimpedance (TEB) has also been adopted to monitor thoracic fluid contents (TFC) in the neonatal population [3–5]. Nevertheless, the impact of an hsPDA on pulmonary fluids during early postnatal phases, which are at greatest risk for RDS development, has not been investigated using these techniques.

This study aimed to evaluate whether the presence of a hsPDA in very preterm infants during the transitional period may influence lung fluids, assessed non-invasively by lung ultrasonography and TEB.

## Methods

The present study involves a sub-analysis of a prospective observational research conducted at the neonatal intensive care units (NICUs) of IRCCS AOU Bologna (Bologna, Italy) and Grande Ospedale Metropolitano Niguarda (Milan, Italy) from May 2020 to January 2022. Infants < 32 weeks’ gestational age (GA) with RDS, defined by an acute respiratory failure with onset within the first 24 h of life, accompanied by evidence of the presence of diffuse, bilateral, irregular lung opacities or infiltrates at chest radiography (if performed) and responsive to lung-recruiting ventilatory strategies and/or to surfactant treatment [6], were included in this sub-analysis. Major congenital malformations, congenital heart disease, perinatal asphyxia, meconium aspiration and pneumothorax were exclusion criteria. Both centers adopted similar protocols for fluid intakes, for the ventilatory management and for surfactant administration in preterm neonates, based on the European Consensus Guidelines on RDS management [7]. The study centers also shared a similar approach for hsPDA pharmacological closure, which was considered if echocardiographic evidence of a hsPDA persisted after 72 h of life or earlier, in case of systemic hypotension requiring inotropic support. The study was conducted in conformity with the Helsinki Declaration principles and approved by the local Ethic Committees (Area Vasta Emilia Centro-AVEC, approval ID:092/201/Oss/AOUBo; Comitato Etico Milano Area3, approval ID:48-12022020). Written, informed consent was obtained from the infants’ parents.

During the first 72 h of life, the enrolled infants underwent daily assessments of lung ultrasonography and echocardiography. Lung ultrasound findings were classified using a validated lung ultrasound score (LUS) assigning the following scores to six pulmonary fields: 0: only A-lines or < 3 B-lines; 1: ≥ 3 B-lines; 2: crowded and confluent B-lines with or without consolidations; 3: significant consolidations [8]. Echocardiographic assessments evaluated the presence of a patent ductus arteriosus and the related echocardiographic features (i.e., diameter, transductal pattern and shunts, left atrial-to-aortic root ratio). A hsPDA was defined by an internal diameter ≥ 1.5 mm or a ductal diameter-to-left pulmonary artery ratio ≥ 0.5 and ≥ 1 of the following criteria: left atrium-to-aortic root ratio ≥ 1.6; ductus flow velocity ≤ 2.5 m/sec or mean transductal pressure gradient ≤ 8 mmHg; left pulmonary artery diastolic flow velocity > 0.2 m/sec; reversed diastolic flow in the descending aorta.

Simultaneously to the sonographic assessments, a 1-h monitoring of TFC and left ventricular output (LVO) was performed using TEB (ICON^®^, Osypka Medical GmbH, Berlin, Germany) with a beat-to-beat sampling frequency. After the recording, the traces were checked to rule out potential artifacts; the signal goodness was also assessed to improve artifact detection as previously described [9]. Averaged TFC and LVO values were indexed for the infants’ weight and used for statistical analysis.

The modality of respiratory support ongoing at each daily assessment was classified as non-invasive (continuous or bilevel positive airway pressure, nasal intermittent positive pressure ventilation) or invasive (conventional or high-frequency oscillatory ventilation via an endotracheal tube) and used as a proxy of RDS severity.

### Statistical analysis

Data distribution was tested using the Shapiro–Wilk test. Categorical variables were summarized as frequencies and percentages. Since the data did not follow normal distribution, numerical variables were summarized as median (interquartile range [IQR]), and between-group comparisons of daily LUS, TFC, and LVO values were performed using the Mann–Whitney U test. To account for repeated measurements on each subject, the independent effect of hsPDA on TFC and LUS, net of such influencing covariates as GA and the modality of respiratory support, was evaluated using generalized estimating equations (GEEs). Since the ductal features and the modality of respiratory support changed over the study period, these variables were handled as time-dependent covariates, and their daily status was included in the GEEs. Statistical analysis was performed using SPSS version 27 (IBM, Armonk, NY). Significance level was set at *p* < 0.05.

## Results

Forty-six preterm infants (median GA 29 [interquartile range, IQR: 27–31] weeks; median birth weight 1099 [IQR 880–1406] g) were included. All the included infants were longitudinally sampled on days 1, 2, and 3. The prevalence of a hsPDA on days 1, 2, and 3 was 26/46 (56.5%), 14/46 (30.4%), and 8/46 (17.4%), respectively; in 4 infants, pharmacological closure of the hsPDA was undertaken on day 3 due to the occurrence of systemic hypotension requiring inotropes. Forty-three (93.5%) out of 46 infants had echocardiographic evidence of a foramen ovale. Invasive ventilation was required by 14/46 infants (30.4%) on day 1, 12/46 (26.1%) on day 2, and 11/46 (23.9%) on day 3. Of the 14 infants who required an invasive respiratory support on day 1, 7 were placed on conventional modalities and 7 on high-frequency oscillatory ventilation with a median MAP of 11 cmH_2_O. All the infants requiring invasive ventilation received endotracheal surfactant before the first LUS and TFC assessment; for this reason, this variable was not included in the multivariable analysis.

As detailed in Table 1, compared to those with a restrictive or a closed duct, infants with a hsPDA showed significantly higher LUS and TFC values as well as a significantly increased LVO on days 1, 2, and 3.

**Table 1:** Comparison of daily lung ultrasound scores (LUS), thoracic fluid contents (TFC), and left ventricular output (LVO) between infants with a haemodynamically significant patent ductus arteriosus (hsPDA) and those with a restrictive or closed duct

	**HsPDA**	**Restrictive or closed duct**	***P*-value**
LUS,** median (IQR)**			
Day 1	10 (7–12)	4 (2–9)	0.004
Day 2	12 (10–12)	5 (2–10)	< 0.001
Day 3	12 (11–12)	7 (2–11)	0.004
TFC (KOhm^−1^),** median (IQR)**			
Day 1	49 (41–57)	36 (29–44)	< 0.001
Day 2	49 (43–57)	40 (31–48)	0.008
Day 3	54 (50–58)	36 (30–44)	< 0.001
LVO (ml/kg/min), **median (IQR)**			
Day 1	300 (282–400)	257 (230–308)	0.014
Day 2	343 (297–398)	279 (230–310)	0.014
Day 3	307 (240–482)	264 (235–323)	0.045

**Fig. 1:**
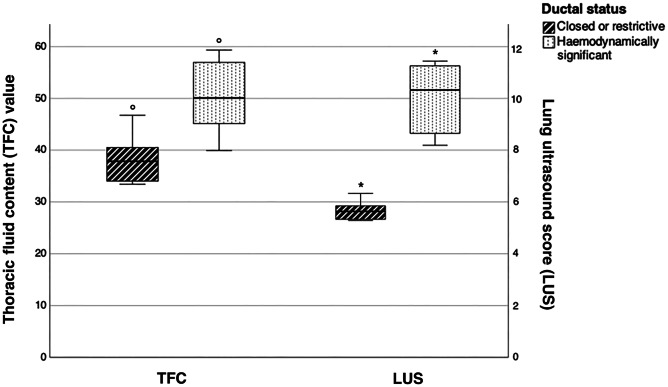
Estimated means of total fluid contents (TFC, left side) and lung ultrasound score (LUS, right side) during the first 3 days of life in relation to the status of the ductus arteriosus. Predictors are fixed at a gestational age of 29 weeks. The bars indicate the 95% confidence interval. The asterisks indicate significant comparisons (*p* < 0.001)

The GEE results confirmed that the presence of a hsPDA significantly increased LUS (β = 2.821 [95%CI 1.315–4.326], *p* < 0.001) and TFC (β = 6.183 [95%CI 2.422–9.944], *p* < 0.001) compared with a restrictive or closed ductus; estimated mean values of both parameters in relation to the ductal status are illustrated in Fig. 1. Ongoing invasive ventilation was also associated with significantly higher LUS (β = 2.194 [95%CI 0.382–4.006], *p* = 0.018) and TFC (β = 4.762 [95%CI 0.234–9.289], *p* = 0.039), whereas an inverse relationship between TFC and GA was observed (β = − 2.099 [95% CI–3.003; − 1.195], *p* < 0.001).

## Discussion

According to the present results, a combined monitoring of lung ultrasonography and TEB in very preterm neonates during the transitional period may effectively detect the effects of a hsPDA on lung fluids. Compared to a closed or restrictive ductus, the presence of a hsPDA was independently associated with significantly higher LUS and TFC values, suggestive of increased lung fluids. As reflected by the increased LVO observed in hsPDA infants, this finding is consistent with the effects of the pulmonary overflow resulting from the excess left-to-right transductal shunting.

Lung ultrasonography has been largely adopted in neonatal care to assess infants with RDS [10, 11]. TEB has also been used to monitor thoracic fluids in RDS neonates and can provide a continuous trend monitoring of their clearance [3–5]. Recently, Savoia et al. combined both techniques to assess LUS and LVO in infants with a hsPDA undergoing surgical ligation and reported a significant LUS and LVO drop after the procedure, consistently with the cessation of pulmonary overflow [12]. To the best of our knowledge, however, this is the first study assessing the impact of a hsPDA on pulmonary fluids during the early postnatal phase using a combined serial monitoring of lung ultrasonography and TEB.

Evidence of lung fluids accumulation in infants with a hsPDA since the early postnatal phase may entail potentially relevant clinical implications. Since the lung fluid accumulation ensuing from the transductal shunting may contribute to the severity of respiratory distress or to its persistence, infants with RDS and echocardiographic evidence of a hsPDA may benefit from a careful balance of fluid intakes during the postnatal transition. A recent survey investigating fluid management in preterm infants with a hsPDA has highlighted a lack of standardization and wide variability among Italian NICUs [13]; in this regard, serial evaluations of LUS and TFC could provide useful information on lung fluid clearance and could be used to monitor the response to fluid-targeted (e.g., intravenous fluid restriction, diuretics) or ventilatory treatment strategies (e.g., increased positive end-expiratory pressure) in these infants. However, whether this information could be used to assist clinicians in the decision-making process on pharmacological hsPDA closure falls outside the aims of our study.

The association between hsPDA persistence and later BPD development has been largely documented [14]. We have previously reported an increased BPD risk in association with TFC values > 41 and LUS > 9 during the transitional period. In the present study, infants with a hsPDA maintained TFC and LUS values above these thresholds throughout the whole monitoring period. Although our findings may suggest a role for early lung fluid overload secondary to hsPDA on later BPD development, this correlation cannot be directly established from the present data.

The small study sample represents needs to be acknowledged as a main limitation. Although similar criteria for fluid management, hsPDA closure, mechanical ventilation, extubation, and surfactant administration were adopted in the two centers, the bicentric nature of the study may limit the generalizability of the present results, which, therefore, need to be validated on larger samples. Moreover, the possible impact of ventilation parameters on LUS and hsPDA could not be assessed in the present study, thus posing a potential bias.

In conclusion, the presence of a hsPDA can have a significant impact on pulmonary fluids since the early postnatal phase. Lung fluid clearance in preterm infants with RDS and a hsPDA can be quantitatively monitored using such non-invasive techniques as lung ultrasounds and TEB, which may add potentially useful information for an individualized management of this population. Nevertheless, further targeted studies are required to validate these findings and to assess their pathophysiological impact on later pulmonary outcomes.

## Data Availability

Data are available from the corresponding author upon reasonable request.

## Abbreviations

CI: Confidence interval
GA: Gestational age
GEEs: Generalized estimating equations
HsPDA: Haemodynamically significant patent ductus arteriosus
IQR: Interquartile range
LUS: Lung ultrasound score
LVO: Left ventricular output
NICUs: Neonatal Intensive Care Units
RDS: Respiratory distress syndrome
TEB: Transthoracic electrical bioimpedance
TFC: Thoracic fluid contents

## Code availability

N/A.
